# Multiple DNA viruses identified in multimammate mouse (*Mastomys natalensis*) populations from across regions of sub-Saharan Africa

**DOI:** 10.1007/s00705-020-04738-9

**Published:** 2020-08-04

**Authors:** Sébastien Calvignac-Spencer, Léonce Kouadio, Emmanuel Couacy-Hymann, Nafomon Sogoba, Kyle Rosenke, Andrew J. Davison, Fabian Leendertz, Michael A. Jarvis, Heinz Feldmann, Bernhard Ehlers

**Affiliations:** 1 P3 “Viral Evolution”, Robert Koch-Institute, Berlin, Germany; 2 LANADA/Central Laboratory for Animal Diseases, Bingerville, Côte d’Ivoire; 3 P3 “Epidemiology of Highly Pathogenic Microorganisms”, Robert Koch-Institute, Berlin, Germany; 4 Faculty of Medicine and Odontostomatology, Malaria Research and Training Center, International Center of Excellence in Research, University of Sciences, Techniques and Technologies of Bamako, Bamako, Mali; 5 Laboratory of Virology, Division of Intramural Research, National Institute of Allergy and Infectious Diseases, National Institutes of Health, Hamilton, MT USA; 6 MRC-University of Glasgow Centre for Virus Research, Glasgow, UK; 7 School of Biomedical Sciences, University of Plymouth, Plymouth, UK; 8 The Vaccine Group Ltd, Plymouth, UK; 9 Division 12 “Measles, Mumps, Rubella, and Viruses Affecting Immunocompromised Patients”, Robert Koch Institut, Berlin, Germany

## Abstract

The multimammate mouse (*Mastomys natalensis; M. natalensis*) serves as the main reservoir for the zoonotic arenavirus Lassa virus (LASV), and this has led to considerable investigation into the distribution of LASV and other related arenaviruses in this host species. In contrast to the situation with arenaviruses, the presence of other viruses in *M. natalensis* remains largely unexplored. In this study, herpesviruses and polyomaviruses were identified and partially characterized by PCR methods, sequencing, and phylogenetic analysis. In tissues sampled from *M. natalensis* populations in Côte d*'*Ivoire and Mali, six new DNA viruses (four betaherpesviruses, one gammaherpesvirus and one polyomavirus) were identified. Phylogenetic analysis based on glycoprotein B amino acid sequences showed that the herpesviruses clustered with cytomegaloviruses and rhadinoviruses of multiple rodent species. The complete circular genome of the newly identified polyomavirus was amplified by PCR. Amino acid sequence analysis of the large T antigen or VP1 showed that this virus clustered with a known polyomavirus from a house mouse (species *Mus musculus polyomavirus 1*). These two polyomaviruses form a clade with other rodent polyomaviruses, and the newly identified virus represents the third known polyomavirus of *M. natalensis*. This study represents the first identification of herpesviruses and the discovery of a novel polyomavirus in *M. natalensis*. In contrast to arenaviruses, we anticipate that these newly identified viruses represent a low zoonotic risk due to the normally highly restricted specificity of members of these two DNA virus families to their individual mammalian host species.

**Electronic supplementary material:**

The online version of this article (10.1007/s00705-020-04738-9) contains supplementary material, which is available to authorized users.

## Introduction

Herpesviruses (order *Herpesvirales*) and polyomaviruses (family *Polyomaviridae*) are double-stranded DNA viruses known to infect many different vertebrate species, including fish, birds and mammals [5, 8]. The multimammate mouse (*Mastomys natalensis; M. natalensis)* is a common rodent belonging to the family Muridae, subfamily Murinae. It occupies a wide geographic range extending across the entirety of sub-Saharan Africa [34]. The natural habitat of *M. natalensis* is equally diverse, with animals well-adapted to agro-ecosystems and cohabitation with humans [24]. *M. natalensis* is the major reservoir for the zoonotic arenavirus Lassa virus (LASV) [31, 38]. Multiple related arenaviruses have also been identified in *M. natalensis*, including Gairo, Luna, Mopeia and Morogoro viruses [19, 34].

Earlier studies identified herpesviruses and polyomaviruses in several rodent species [11, 14], including two polyomaviruses in *M. natalensis* corresponding to an alphapolyomavirus [14] and a betapolyomavirus [35]. However, *M. natalensis* has never been examined for the presence of herpesviruses. In the present study, we analysed tissues from *M. natalensis* collected in Côte d*'*Ivoire (CI) in 2014 and in Mali in 2017 for the presence of herpesviruses (CI, Mali) and polyomaviruses (Mali). Our results show that *M. natalensis* carries multiple herpesviruses, predominantly belonging to the subfamily *Betaherpesvirinae* but also to the subfamily *Gammaherpesvirinae*. We also identified a novel polyomavirus, representing the third polyomavirus identified in *M. natalensis*.

## Materials and methods

### Sample collection

*M. natalensis* were live-captured, deeply anesthetized with isoflurane, bled by cardiac puncture, and euthanized by cervical dislocation. All procedures on live animals were conducted in compliance with the applicable institutional and national guidelines for use and handling of animals. Tissues were immediately flash frozen and stored at -80 °C or below. Samples were confirmed to originate from *M. natalensis* by *cytochrome b* PCR, sequencing, and a BLAST search of the GenBank database [25].

### PCR methods

**Fig. 1:**
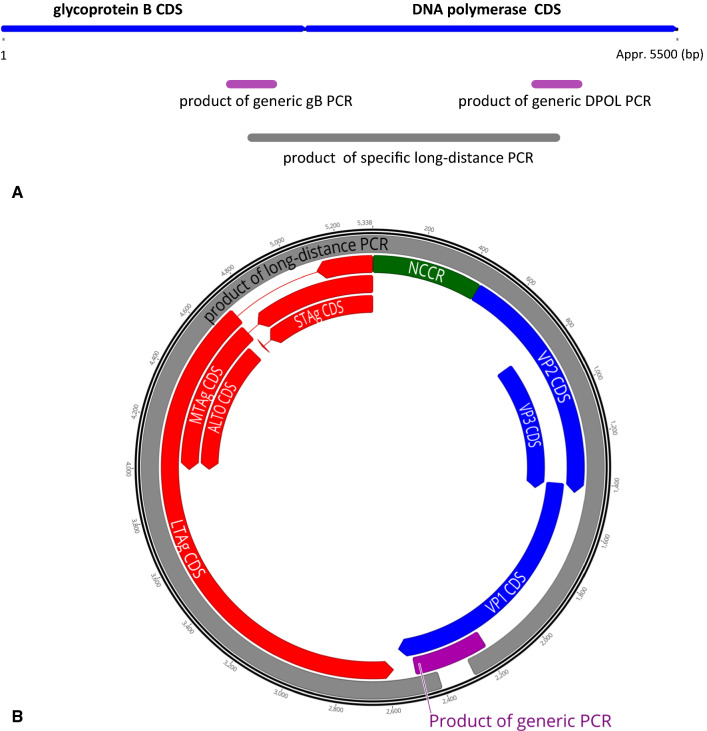
Amplification of herpesvirus and polyomavirus sequences. **(A)** Sequences of the herpesvirus DNA polymerase (DPOL) and glycoprotein B (gB) CDSs (depicted schematically by blue bars) were amplified by generic DPOL and gB PCR. The respective products are depicted by magenta bars. The sequence between these regions of DPOL and gB was then amplified by long-distance PCR (grey bar). **(B)** The genome of Mastomys natalensis polyomavirus 3 was amplified by generic PCR (magenta bar) targeting the VP1 CDS. The remaining part of the genome was amplified by inverse long-distance PCR using ‘back-to-back’ primers (grey bar). The CDSs in the early region are depicted by red arrows, those in the late region by blue arrows, and the NCCR by a green bar

DNA was extracted and tested for the presence of herpesviruses by a generic nested PCR targeting a region of the herpesvirus DNA polymerase (DPOL) gene (Fig. 1A) as described previously [11]. Glycoprotein B (gB) coding sequences of viruses of the subfamilies *Betaherpesvirinae* and *Gammaherpesvirinae* were also amplified using a generic nested PCR [11]. For testing for the presence of polyomaviruses, a generic polyomavirus PCR (Fig. 1B) was performed that targets the major capsid VP1 gene of mammalian polyomaviruses [26, 39]. Specific long-distance (LD)-PCR (Fig. 1A and B) was carried out using a TaKaRa Ex Taq PCR Kit (Clontech, California, USA) according to manufacturer’s instructions.

### Availability of data

The novel sequences were deposited in the GenBank database under accession numbers MN417224- MN417229.

### Phylogenetic analysis

For phylogenetic analysis, reference viruses were selected representing all currently recognized species in the family or subfamily, as well as additional viruses that represented distinct viral lineages discussed in the literature but not yet integrated into the official taxonomy (sensu International Committee on Taxonomy of Viruses [ICTV]). This represented 111, 21, and 40 polyoma-, betaherpes-, and gammaherpesviruses, respectively. The coding sequences (CDSs) of polyomavirus large T antigen (LTAg) and VP1 or herpesvirus DPOL and gB were extracted using Geneious v11.1.5 [23]. The CDSs were translated into amino acid sequences, which were aligned using MUSCLE [9] as implemented in Seaview v4 [16]. Blocks of conserved amino acids were then selected using Gblocks, with selection criteria for a less stringent selection as implemented in Seaview [41].

For each dataset, a maximum-likelihood (ML) analysis was carried out using PhyML v3 with smart model selection (PhyML-SMS) and the Bayesian information criterion and a tree search using subtree pruning and regrafting [16, 18, 27]. Branch robustness was estimated using Shimodaira-Hasegawa-like approximate likelihood ratio tests (SH-like aLRT) [1]. The polyomavirus and gammaherpesvirus ML trees were rooted with TempEst v1.5 by minimizing the variance of root-to-tip distances [36]; the betaherpesvirus ML trees were rooted using roseolovirus outgroups. Bayesian Markov chain Monte Carlo (BMCMC) analysis was then carried out using BEAST v1.10.4 [40]. For each alignment, the amino acid substitution model identified by PhyML-SMS was used with an uncorrelated relaxed clock (lognormal) model and a speciation model (birth–death) as a tree prior. The output of multiple BMCMC runs was examined for convergence and appropriate sampling of the posterior using Tracer v1.7.1 [37] before being merged using LogCombiner v1.10.4 (distributed with BEAST). The maximum-clade-credibility tree (MCC tree) was identified from the posterior set of trees (PST) and annotated with TreeAnnotator v1.10.4 (also distributed with BEAST). Branch robustness was estimated based on posterior probability values in the PST. The final amino acid sequence alignments comprised 264 (VP1), 523 (LTAg), 615 (betaherpesvirus DPOL), 384 (betaherpesvirus gB), 627 (gammaherpesvirus DPOL) and 284 (gammaherpesvirus gB) amino acid residues.

## Results

DNA was extracted from 72 archived *M. natalensis* spleen samples (Mali) and 103 M*. natalensis* lung samples (CI; these samples had already been tested for the presence of LASV [25] and polyomaviruses [14])*.* DNA samples were tested for the presence of herpesviruses by generic nested DPOL PCR, and PCR products of the predicted size were sequenced. As indicated by BLAST analysis, 45 samples were herpesvirus-positive. The sequences from 39 samples matched members of the subfamily *Betaherpesvirinae*, and those from six samples matched members of the subfamily *Gammaherpesvirinae* (Table 1). Consistent with the lack of alphaherpesviruses in other murids, no alphaherpesviruses were detected. The betaherpesvirus sequences originated from four distinct viruses that were tentatively named "Mastomys natalensis cytomegalovirus 1 to 4" (MnatCMV1-4) on the basis of phylogenetic clustering with members of this genus. The five gammaherpesvirus sequences were identical and represented the same virus, which was tentatively named "Mastomys natalensis rhadinovirus 1" (MnatRHV1).

**Table 1:** Herpesviruses in *Mastomys natalensis*

Herpesvirus	Conservation (% identity vs MnatCMV1)^a^	Frequency in CI	Frequency in Mali
MnatCMV1	100	8/103 (8%)	0/72 (0%)
MnatCMV2	87	5/103 (5%)	0/72 (0%)
MnatCMV3	61	13/103 (13%)	7/72 (10%)
MnatCMV4	69	4/103 (4%)	2/72 (3%)
MnatRHV1		6/103 (3%)	0/72 (0%)

^a^Based on pairwise alignments of partial DPOL nucleic acid sequences (178 bp)

The partial DPOL nucleic acid sequences of MnatCMV1-4 revealed pairwise identities of 69–87% (Table 1). On the basis of BLAST analysis, these viruses were most similar to known rodent cytomegaloviruses from gerbil (*Dipodillus* spp.) herpesvirus (MnatCMV1), Malayan field rat (*Rattus tiomanicus)* cytomegalovirus 1 (MnatCMV2), and wood mouse (*Apodemus sylvaticus)* cytomegalovirus 1 (MnatCMV3 and 4). The partial DPOL sequence of MnatRHV1 was most similar to that of a gammaherpesvirus of the house mouse (Mus musculus rhadinovirus 1; 55% identity). MnatCMV1, MnatCMV2, and MnatRHV1 were detected only in animals from CI, whereas MnatCMV3 and 4 were present in animals from both countries. In terms of the divergence of *M. natalensis* throughout these geographic regions, Mali and CI are believed to be represented by a single phylogenetic group (named A-I; [7]). The differences in virus distribution may therefore reflect real geographical differences or may be resolved by more-extensive sampling. Overall, herpesvirus frequency in the *M. natalensis* lungs (CI) and spleens (Mali) was 34% and 13%, respectively. In the spleen samples from Mali, only MnatCMV3 and MnatCMV4 were detected, possibly suggesting distinct tissue tropism of individual viruses for lung compared to spleen.

Next, betaherpesvirus- and gammaherpesvirus-positive DNAs were tested by generic PCR assays (Fig. 1A) targeting the gB coding sequences of members of the subfamilies *Betaherpesvirinae* and *Gammaherpesvirinae*, respectively, with sequencing of amplified products. Glycoprotein B sequences were identified for MnatCMV1 (two samples), MnatCMV3 (five samples) and MnatRHV1 (three samples), but not for MnatCMV2 and 4. Based on the partial DPOL and gB sequences of MnatCMV1, MnatCMV3 and MnatRHV1, sequence-specific primer pairs were selected for each virus from the respective gB and DPOL sequences and used in LD-PCR (Fig. 1A). This resulted in amplification of a 3.2-kilobase pair (kbp) sequence. Assembly with the initial gB and DPOL sequences resulted in a 3.4-kbp contiguous sequence stretching from the 3´ region of gB to the 5´ region of DPOL. This approach was successful for two samples positive for MnatCMV3 and three samples containing MnatRHV1, but not for any of the MnatCMV1-containing samples.

Spleen DNA samples from Mali were also tested for the presence of polyomaviruses by generic PyV PCR (Fig. 1B). One sample was positive, with the sequence being identified by BLAST analysis as originating from a new polyomavirus distinct from the two known polyomaviruses of *M. natalensis*. Specific nested ‘back-to-back’ primers were selected for the VP1 sequence, and a 5.2-kbp product was amplified by LD-PCR (Fig. 1B), followed by sequencing using classical ‘primer-walking’. The sequences of the initial generic PCR product and the LD-PCR product were then used to assemble a contiguous circular sequence, resulting in the generation of a complete polyomavirus genome of 5338 bp. Open reading frame analysis using Geneious 11.1.5 software showed the genome to exhibit a typical polyomavirus genome organization: (1) an early region encoding large, middle, and small T antigen CDSs and (2) a late region on the opposite strand encoding the VP1, VP2 and VP3 capsid proteins. Early and late regions were separated by a non-coding control region (NCCR). The genome also contained a CDS encoding a putative ALTO protein [6] of 221 amino acid residues (Fig. 1B).

Two *M. natalensis* polyomaviruses had been identified previously: (1) a more distantly related one, named Mastomys polyomavirus (species *Mastomys natalensis polyomavirus 1,* genus *Betapolyomavirus*; accession number AB588640 [35]), from animals in Zambia and (2) a more closely related alphapolyomavirus, named M. natalensis polyomavirus 2 (accession number MG701350) [14], from animals in CI. We therefore tentatively named the new polyomavirus identified in the Mali animals as "Mastomys natalensis polyomavirus 3" (MnatPyV3). The full genome sequence of MnatPyV3 revealed pairwise identity of 46% and 55% to those of MnatPyV1 and MnatPyV2, respectively, and was most similar (66% identity) to murine polyomavirus (species *Mus musculus polyomavirus 1*, genus *Alphapolyomavirus*; accession number AF442959).

As only a few polyomaviruses, all alphapolyomaviruses, have been shown to encode a middle T antigen or an ALTO protein, we compared the predicted middle T antigen CDS of MnatPyV3 with that of its closest relative, murine PyV. We found that the splice donor and acceptor sites are conserved in sequence and position and that the encoded proteins have 61% amino acid sequence identity. The putative ALTO CDS of MnatPyV3 is similar in length and genomic position to the ALTO CDS of Merkel cell polyomavirus (MCPyV), with a hydrophobic motif at its C-terminus similar to that of MCPyV [6]. These comparisons add strength to the prediction that MnatPyV3 encodes a middle T antigen and/or an ALTO protein.

**Fig. 2:**
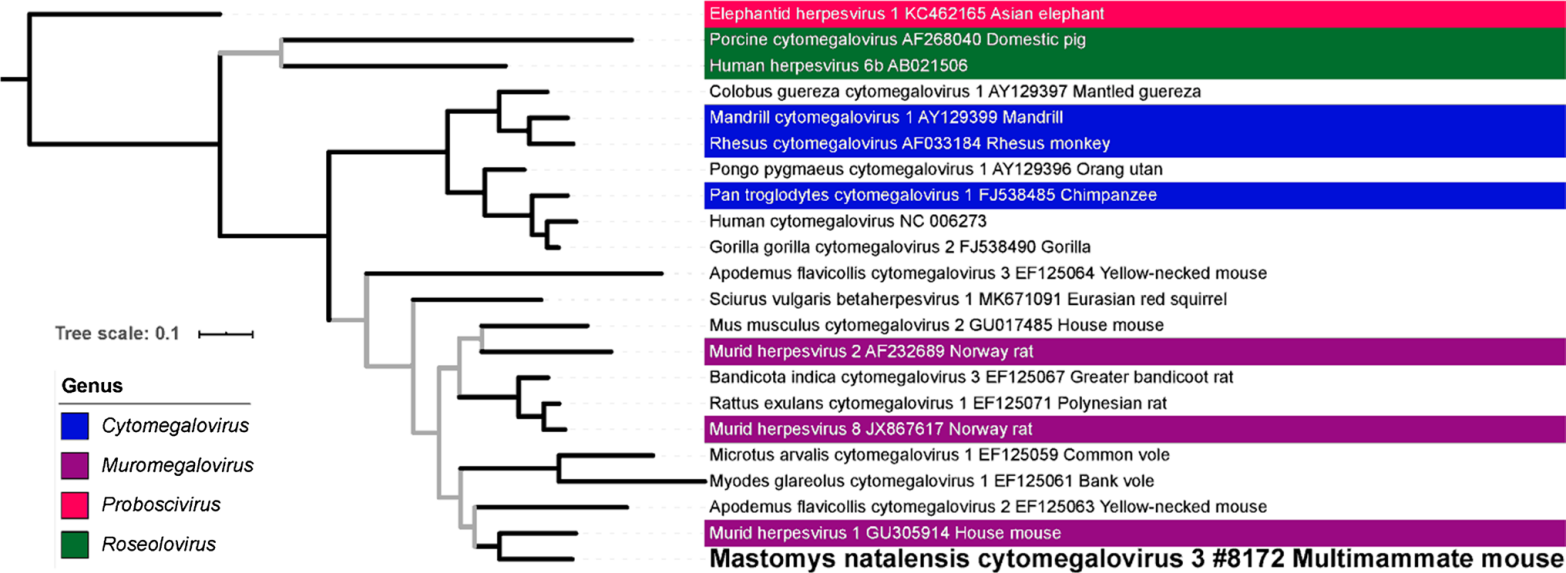
Maximum-likelihood tree of betaherpesviruses based on blocks of conserved amino acids in glycoprotein B. Betaherpesviruses are denoted by Latin taxonomic name or common name, followed by GenBank accession number and host common name. Denotation of the novel Mastomys natalensis cytomegalovirus 3 identified herein is in bold font and also includes the sample ID. For ICTV-recognized virus species, genera are indicated by the colored background of the virus name. Grey branches are relatively weakly supported with posterior probability values < 0.95

**Fig. 3:**
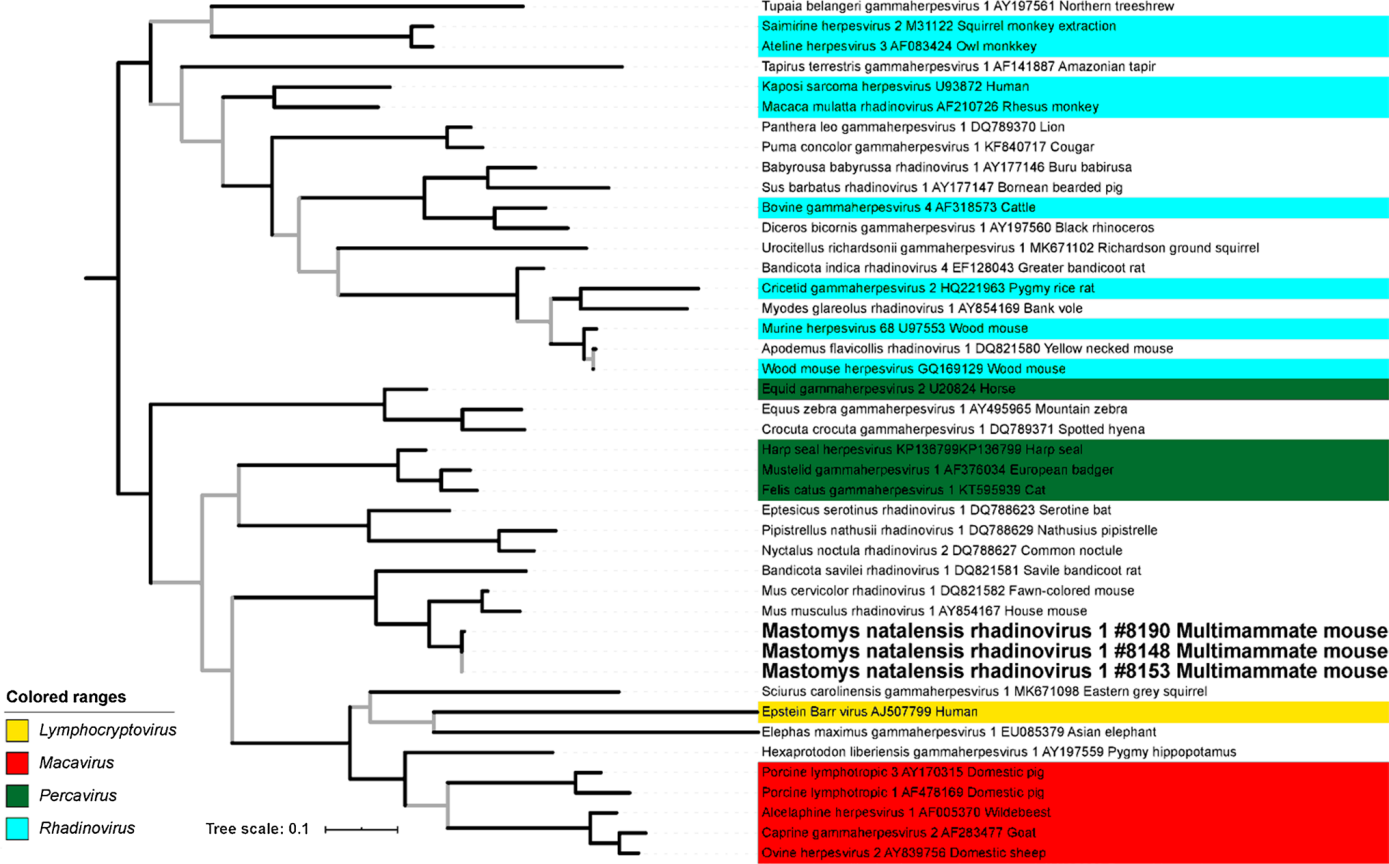
Maximum-likelihood tree of gammaherpesviruses based on blocks of conserved amino acids in glycoprotein B. For explanation see legend of Fig. 2

To investigate the evolutionary position of the novel herpesviruses, we carried out phylogenetic analysis on viruses for which the 3.4-kbp sequence was available, namely, MnatCMV3 and MnatRHV1. ML and BMCMC analyses performed on MnatCMV3 and known representatives of the subfamily *Betaherpesvirinae* using an alignment of gB amino acid sequences [11] suggested that MnatCMV3 is a member of a monophyletic group of rodent CMVs that comprises three species assigned by the ICTV to the genus *Muromegalovirus* (Fig. 2; Online Resource 1). Phylogenetic analysis of an alignment of gB sequences of viruses representing the subfamily *Gammaherpesvirinae* showed that MnatRHV1 is in a sister taxon to a group of rodent gammaherpesviruses (Fig. 3; Online Resource 2). This group was described previously as comprising rhadinoviruses and includes the rhadinovirus of house mouse (Mus musculus rhadinovirus 1) [11]. However, these viruses did not form a monophyletic group with members of recognized rhadinovirus species [30], at least using the tree rooting that we employed (minimization of root-to-tip distance variance with TempEst [36]). Phylogenetic analysis of DPOL alignments of the beta- and gammaherpesviruses yielded similar results (Online Resource 3 and Online Resource 4 [betaherpesviruses] and Online Resource 5 and Online Resource 6 [gammaherpesviruses]).

**Fig. 4:**
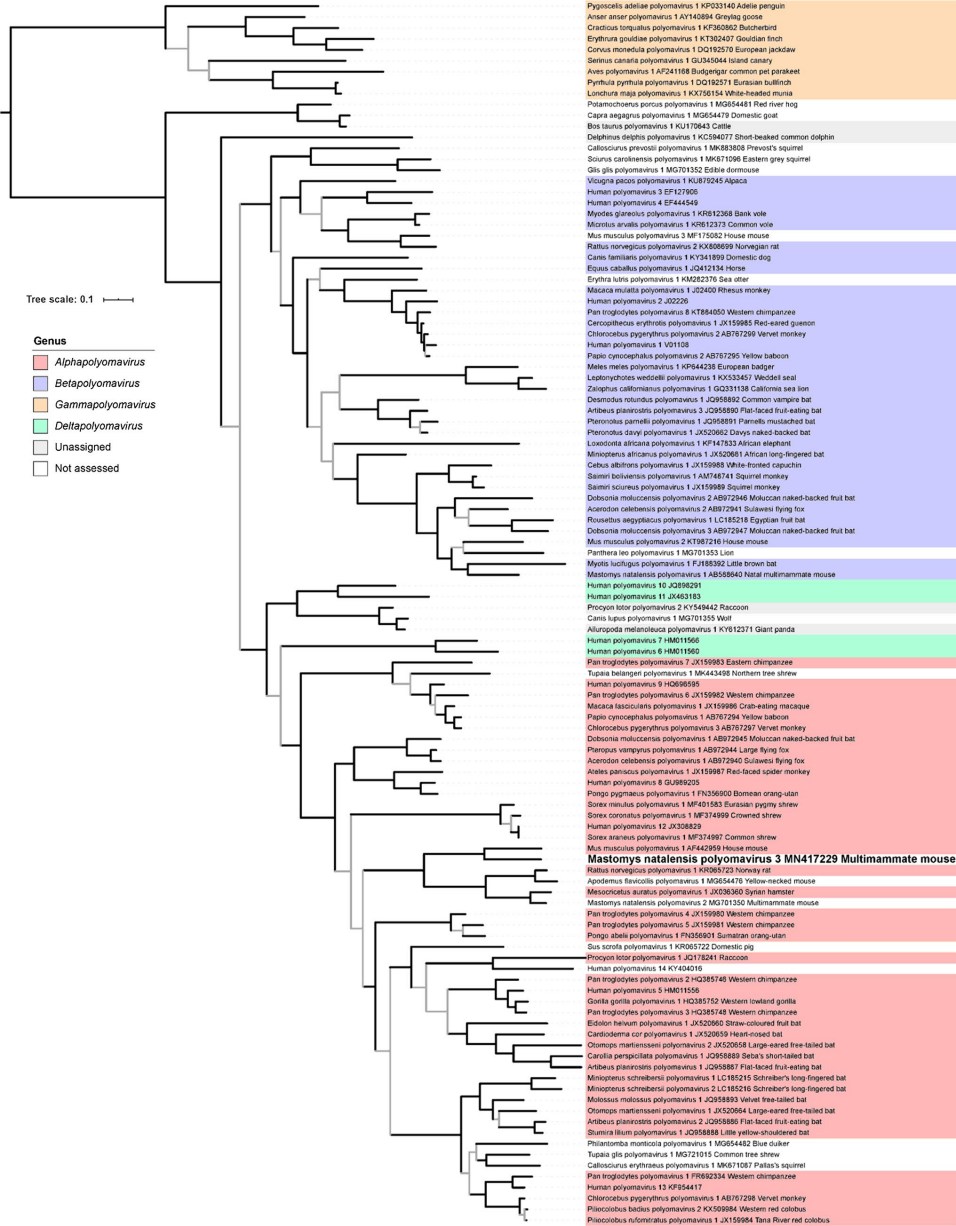
Maximum-likelihood tree of polyomaviruses based on blocks of conserved amino acids in the large T antigen. Polyomavirus nomenclature follows the recommendations of the ICTV *Polyomaviridae* Study Group using the Latin binomials of their hosts followed by a serial number; GenBank accession numbers and vernacular names of the host are also given. Virus genera are indicated by colored background. Mastomys natalensis cytomegalovirus 3 identified in this study is identified in bold font. Grey branches are relatively weakly supported with posterior probability values < 0.95

Phylogenetic analysis based on the amino acid sequences of LTAg of MnatPyV3 and representatives of all polyomavirus species currently recognized by the ICTV showed that MnatPyV3 is a single member of a sister taxon to the murine polyomavirus (species *Mus musculus polyomavirus 1*). The clade formed by these two viruses is itself in sistership with a monophyletic group comprising only rodent alphapolyomaviruses, including rat (species *Rattus norvegicus polyomavirus 1*) and hamster (species *Mesocricetus auratus polyomavirus 1*) polyomaviruses and another polyomavirus infecting *M. natalensis*, MnatPyV2 (Fig. 4; Online Resource 7). Phylogenetic analysis of a VP1 alignment of the same polyomaviruses supported a similar topology (Online Resource 8 and Online Resource 9).

## Discussion

*M. natalensis* is highly adapted to close cohabitation with humans. This characteristic, combined with a high frequency and extensive geographic range, makes the rodent a high-value species in terms of potential for zoonotic microbial flow to humans. *M. natalensis* carries arenaviruses throughout sub-Saharan Africa and is known to serve as a continuing source of human LASV infection in West African countries, mainly Sierra Leone, Guinea, Liberia, and Nigeria. This has led to the intensive study of LASV and other arenaviruses in this rodent species [17, 34]. However, beyond arenaviruses little is known regarding the microbes that infect these animals. *M. natalensis* has been reported to be a reservoir species of *Borrelia crocidurae* [3], the etiological agent of relapsing fever, and is also known to carry *Yersinia pestis*. The latter pathogen-host interaction is believed to play a key role in the plague cycle in southern Africa [22]. Similarly, a papillomavirus (Mastomys natalensis papillomavirus) has been identified in *M. natalensis* and is believed to be causally associated with a high incidence of cutaneous skin tumors in these animals [42]. Our results are compatible with the notion that the herpesviruses and polyomaviruses identified in *M. natalensis* arose from lineages of viruses closely associated with rodents. The occurrence of multiple herpesviruses and polyomaviruses in *M. natalensis* is not surprising, as infection with members of multiple species of a DNA virus family is common in all host species that have been examined in any detail (e.g. [10–13, 39]). It is tempting to interpret this pattern as suggesting that these viruses co-diverged with their hosts and are *M. natalensis*-specific, since co-divergence is an important process in shaping herpesvirus and polyomavirus evolution [2, 4, 14, 15, 28, 33]. Therefore, despite the commensality, abundance, and extensive geographic range of this rodent species, we do not expect these DNA viruses to represent a major zoonotic threat.

CMVs are showing promise as the basis of a generation of host-specific vaccine vectors [20, 21, 29, 32, 43]. In this context, our study opens the way to developing MnatCMVs to target zoonotic pathogens in *M. natalensis*. As a component of an ongoing multi-institutional study, we have since isolated several infectious MnatCMVs and sequenced their genomes. We plan to clone these genomes as infectious bacterial artificial chromosomes, thereby enabling the development of a novel scalable vaccine platform for combating zoonotic pathogens in this important reservoir host.

## Conclusions

In addition to a novel polyomavirus, this study represents the first identification of herpesviruses in *M. natalensis*. In contrast to the arenaviruses commonly found in this rodent species, we anticipate that these newly identified viruses represent a low zoonotic risk due to the normally highly restricted specificity of DNA viruses such as polyomaviruses and herpesviruses to their individual mammalian host species.

## Electronic supplementary material

Below is the link to the electronic supplementary material.

ESM 1 Maximum-clade-credibility tree analysis of betaherpesviruses based on glycoprotein B. Phylogenetic relationships of betaherpesviruses, including the novel Mastomys natalensis cytomegalovirus 3, based on blocks of conserved amino acids in the glycoprotein B sequence. For further details, see the legend of Figure 2. (PPTX 98 kb)

ESM 2 Maximum-clade-credibility tree analysis of gammaherpesviruses based on glycoprotein B. Phylogenetic relationships of gammaherpesviruses, including the novel Mastomys natalensis rhadinovirus 1, based on blocks of conserved amino acids in the glycoprotein B sequence. For further details, see the legend of Figure 2. (PPTX 112 kb)

ESM 3 Maximum likelihood tree analysis of betaherpesviruses based on DNA polymerase. Phylogenetic relationships of betaherpesviruses, including the novel Mastomys natalensis cytomegaloviruses, based on blocks of conserved amino acids in the DNA polymerase sequence. For further details, see the legend of Figure 2. (PPTX 99 kb)

ESM 4 Maximum-clade-credibility tree analysis of betaherpesviruses based on DNA polymerase. Phylogenetic relationships of betaherpesviruses, including the novel Mastomys natalensis cytomegaloviruses, based on blocks of conserved amino acids in the DNA polymerase sequence. For further details, see the legend of Figure 2. (PPTX 87 kb)

ESM 5 Maximum-likelihood tree analysis of gammaherpesviruses based on DNA polymerase. Phylogenetic relationships of gammaherpesviruses, including the novel Mastomys natalensis rhadinovirus 1, based on blocks of conserved amino acids in the DNA polymerase sequence. For further details, see the legend of Figure 2. (PPTX 118 kb)

ESM 6 Maximum clade credibility tree analysis of gammaherpesviruses based on DNA polymerase. Phylogenetic relationships of gammaherpesviruses, including the novel Mastomys natalensis rhadinovirus 1, based on blocks of conserved amino acids in the DNA polymerase sequence. For further details, see the legend of Figure 2. (PPTX 109 kb)

ESM 7 Maximum-clade-credibility tree analysis of polyomaviruses based on the large T antigen. Phylogenetic relationships of polyomaviruses, including the novel Mastomys natalensis polyomavirus 3, based on blocks of conserved amino acids in the large T sequence. For further details, see the legend of Figure 4. (PPTX 171 kb)

ESM 8 Maximum-likelihood tree analysis of polyomaviruses based on VP1. Phylogenetic relationships of polyomaviruses, including the novel Mastomys natalensis polyomavirus 3, based on blocks of conserved amino acids in the VP1 sequence. For further details, see the legend of Figure 4. (PPTX 127 kb)

ESM 9. Maximum-clade-credibility tree analysis of polyomaviruses based on VP1. Phylogenetic relationships of polyomaviruses, including the novel Mastomys natalensis polyomavirus 3, based on blocks of conserved amino acids in the VP1 sequence. For further details, see the legend of Figure 4. (PPTX 170 kb)

## Abbreviations

BMCMC: Bayesian Markov chain Monte Carlo
CI: Côte d'Ivoire
DPOL: DNA polymerase
LASV: Lassa virus
gB: Glycoprotein B
LD-PCR: Long-distance PCR
LTAg: Large T antigen
MCPyV: Merkel cell polyomavirus
ML: Maximum likelihood
*M. natalensis*: *Mastomys natalensis*
MnatCMV: Mastomys natalensis cytomegalovirus
MnatPyV: Mastomys natalensis polyomavirus
MnatRHV: Mastomys natalensis rhadinovirus
PhyML-SMS: Maximum-likelihood analysis using PhyML v3 with smart model selection
PST: Posterior set of trees
PyV: Polyomavirus
SH-like aLRT: Shimodaira-Hasegawa-like approximate likelihood ratio test
VP1: Virion protein 1
