# Supplementary figures and images for: Multiple DNA viruses identified in multimammate mouse (Mastomys natalensis) populations from across regions of sub-Saharan Africa
Source: Arch Virol. 2020 Aug 4;165(10):2291–9. doi: 10.1007/s00705-020-04738-9 (PMC7497350; doi:10.1007/s00705-020-04738-9)

## Slide 1
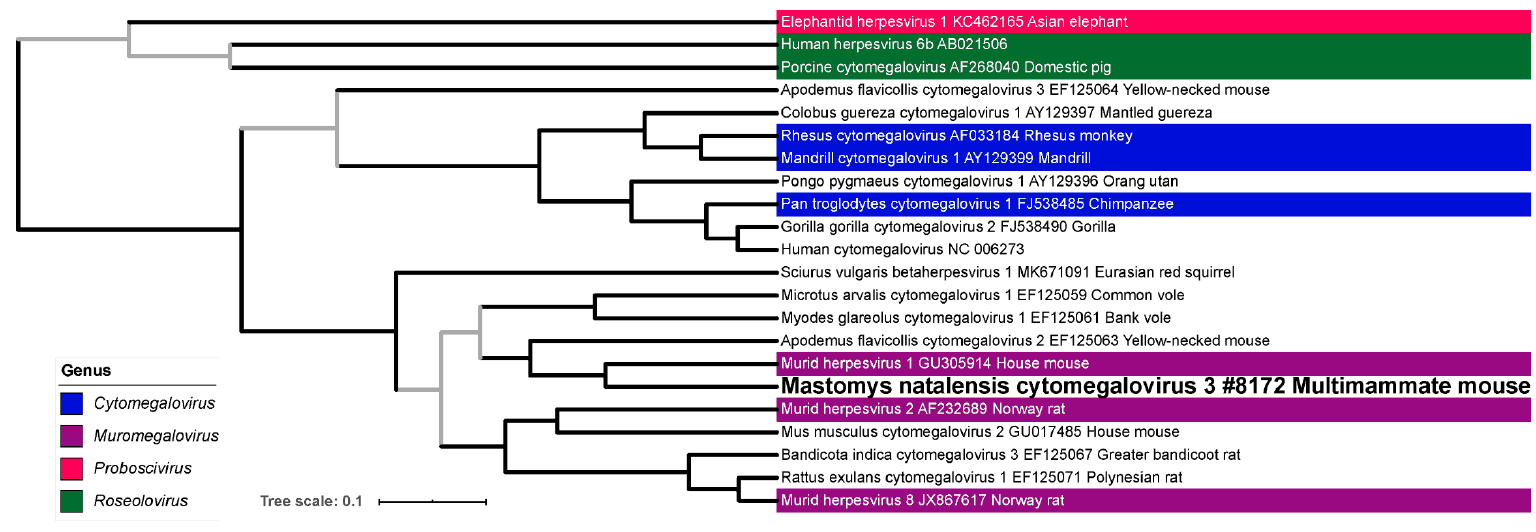

Supplement: Supplementary file 1 — ESM 1 Maximum-clade-credibility tree analysis of betaherpesviruses based on glycoprotein B. Phylogenetic relationships of betaherpesviruses, including the novel Mastomys natalensis cytomegalovirus 3, based on blocks of conserved amino acids in the glycoprotein B sequence. For further details, see the legend of Figure 2. (PPTX 98 kb) [file 705_2020_4738_MOESM1_ESM.pptx]

## Slide 1
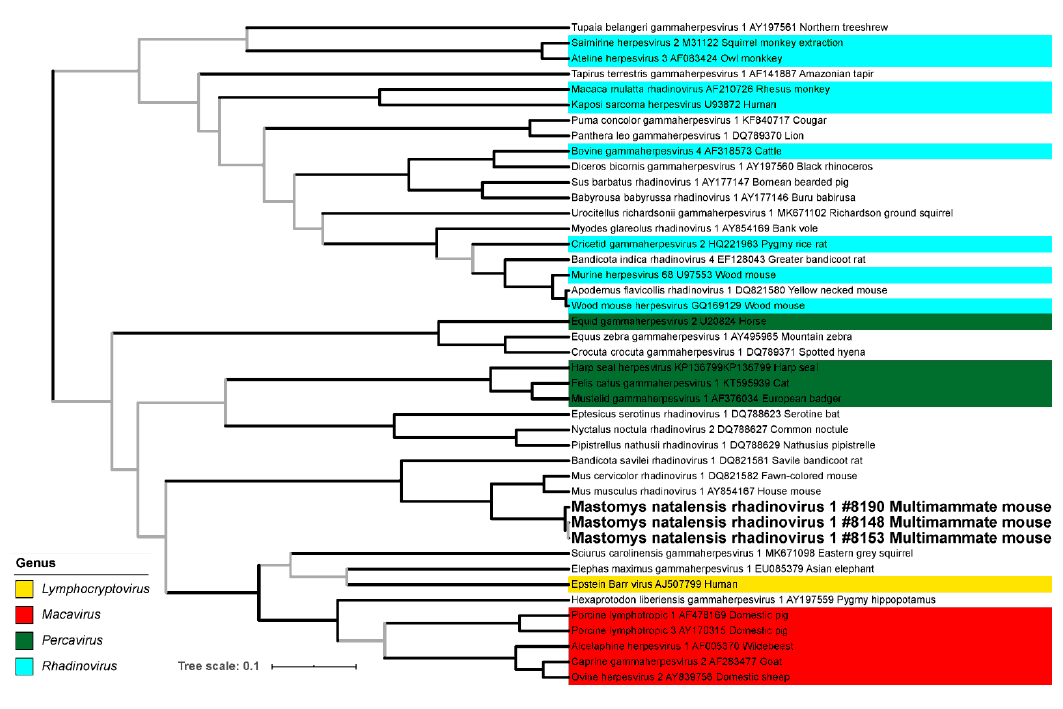

Supplement: Supplementary file 2 — ESM 2 Maximum-clade-credibility tree analysis of gammaherpesviruses based on glycoprotein B. Phylogenetic relationships of gammaherpesviruses, including the novel Mastomys natalensis rhadinovirus 1, based on blocks of conserved amino acids in the glycoprotein B sequence. For further details, see the legend of Figure 2. (PPTX 112 kb) [file 705_2020_4738_MOESM2_ESM.pptx]

## Slide 1
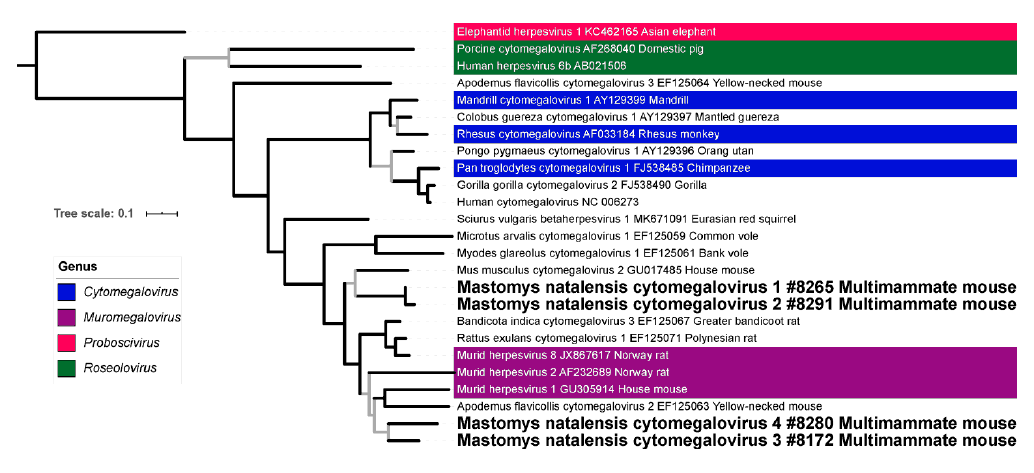

Supplement: Supplementary file 3 — ESM 3 Maximum likelihood tree analysis of betaherpesviruses based on DNA polymerase. Phylogenetic relationships of betaherpesviruses, including the novel Mastomys natalensis cytomegaloviruses, based on blocks of conserved amino acids in the DNA polymerase sequence. For further details, see the legend of Figure 2. (PPTX 99 kb) [file 705_2020_4738_MOESM3_ESM.pptx]

## Slide 1
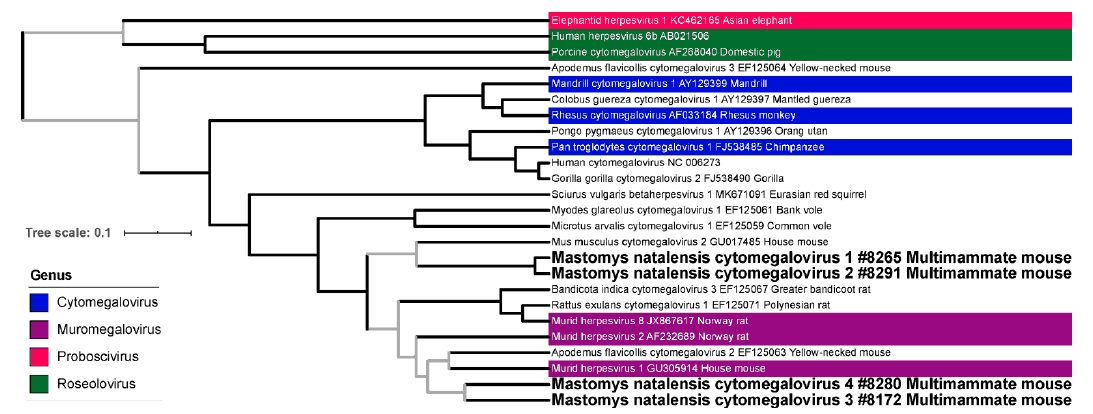

Supplement: Supplementary file 4 — ESM 4 Maximum-clade-credibility tree analysis of betaherpesviruses based on DNA polymerase. Phylogenetic relationships of betaherpesviruses, including the novel Mastomys natalensis cytomegaloviruses, based on blocks of conserved amino acids in the DNA polymerase sequence. For further details, see the legend of Figure 2. (PPTX 87 kb) [file 705_2020_4738_MOESM4_ESM.pptx]

## Slide 1
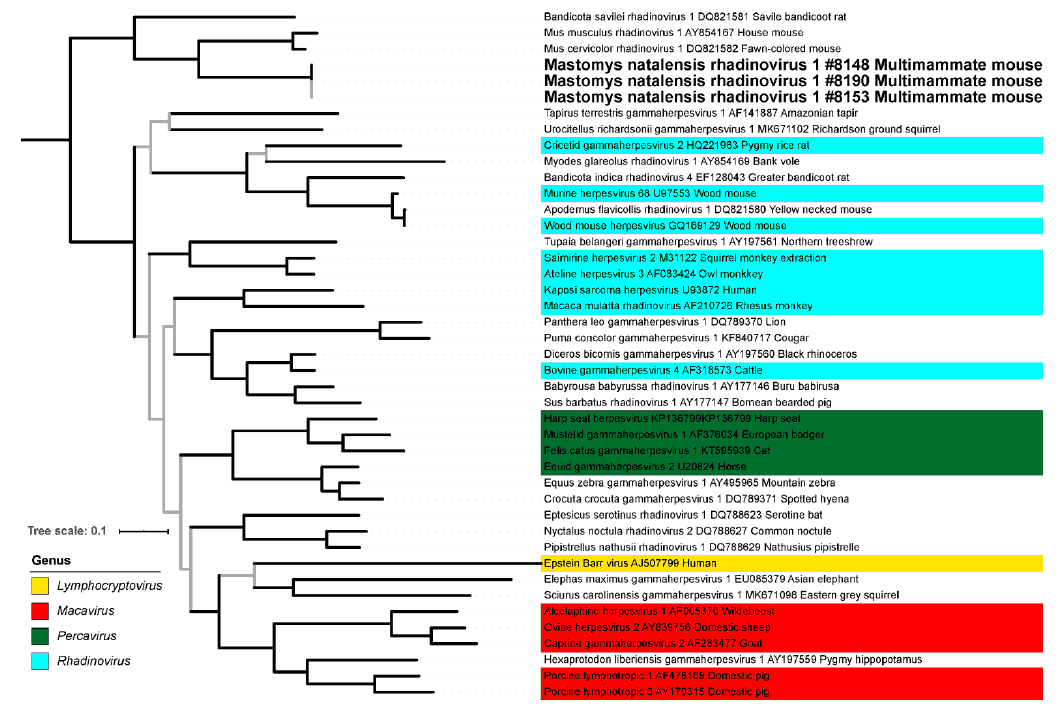

Supplement: Supplementary file 5 — ESM 5 Maximum-likelihood tree analysis of gammaherpesviruses based on DNA polymerase. Phylogenetic relationships of gammaherpesviruses, including the novel Mastomys natalensis rhadinovirus 1, based on blocks of conserved amino acids in the DNA polymerase sequence. For further details, see the legend of Figure 2. (PPTX 118 kb) [file 705_2020_4738_MOESM5_ESM.pptx]

## Slide 1
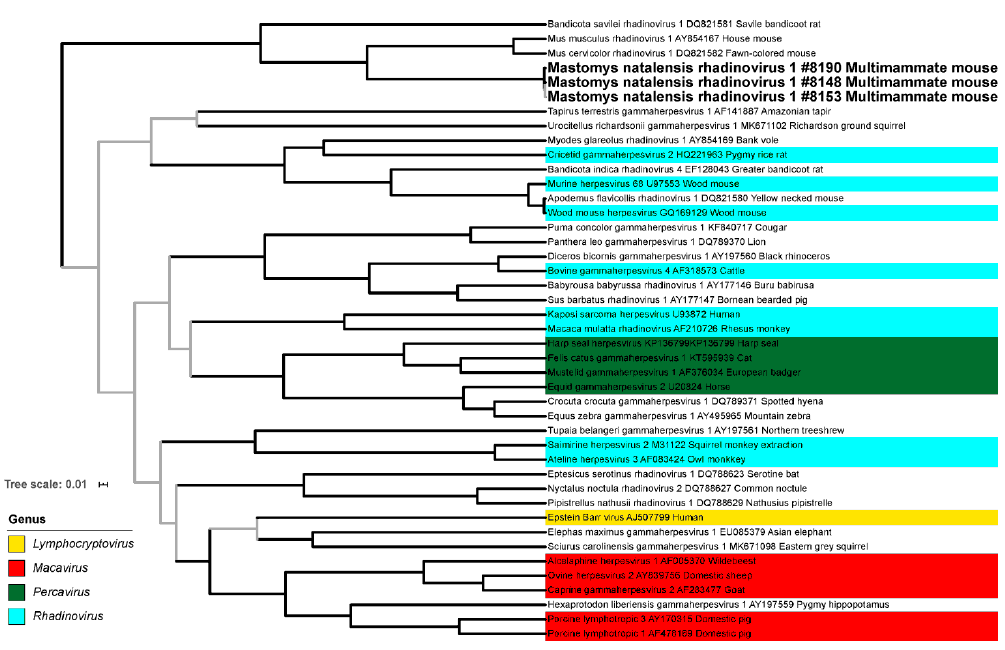

Supplement: Supplementary file 6 — ESM 6 Maximum clade credibility tree analysis of gammaherpesviruses based on DNA polymerase. Phylogenetic relationships of gammaherpesviruses, including the novel Mastomys natalensis rhadinovirus 1, based on blocks of conserved amino acids in the DNA polymerase sequence. For further details, see the legend of Figure 2. (PPTX 109 kb) [file 705_2020_4738_MOESM6_ESM.pptx]

## Slide 1
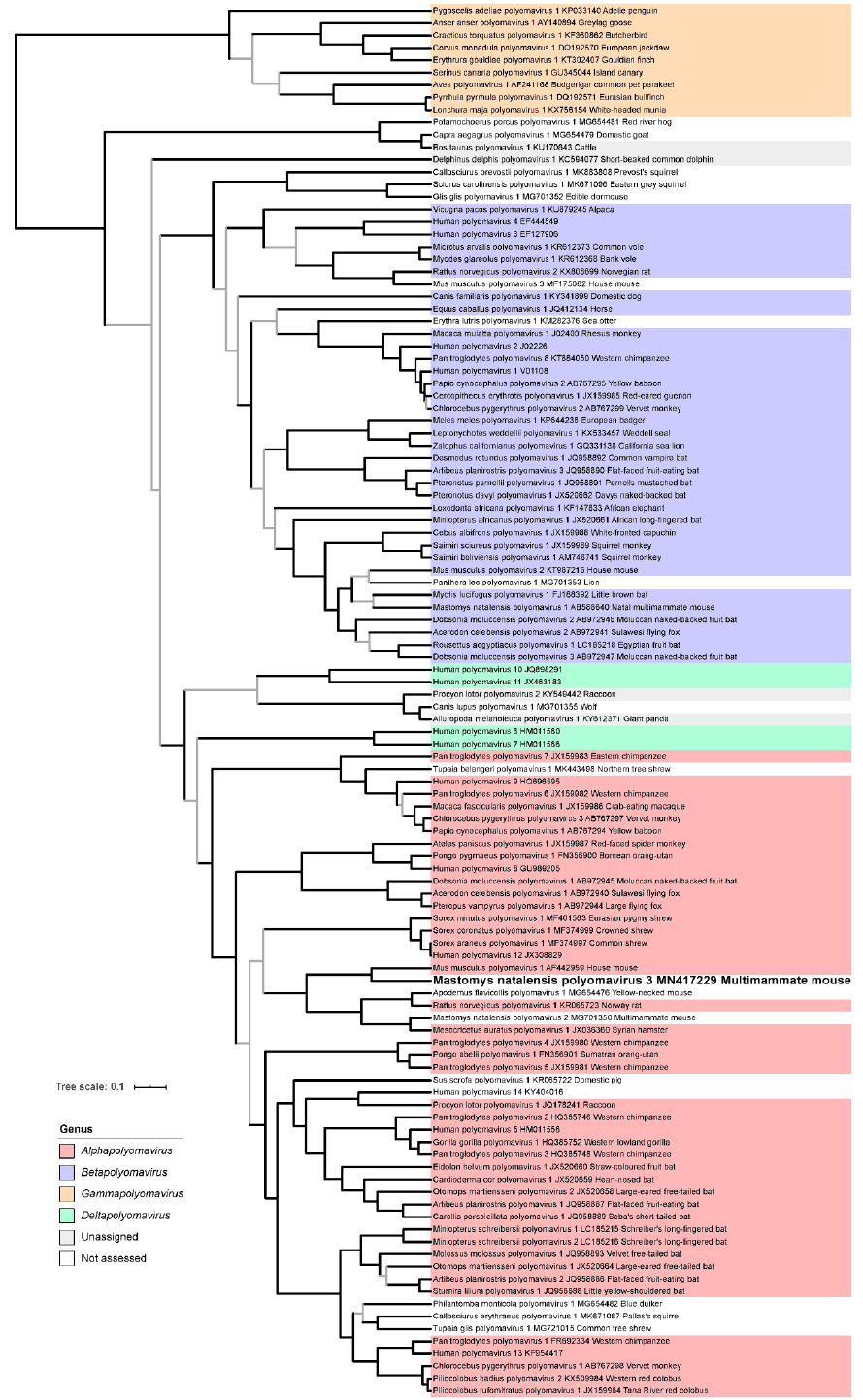

Supplement: Supplementary file 7 — ESM 7 Maximum-clade-credibility tree analysis of polyomaviruses based on the large T antigen. Phylogenetic relationships of polyomaviruses, including the novel Mastomys natalensis polyomavirus 3, based on blocks of conserved amino acids in the large T sequence. For further details, see the legend of Figure 4. (PPTX 171 kb) [file 705_2020_4738_MOESM7_ESM.pptx]

## Slide 1
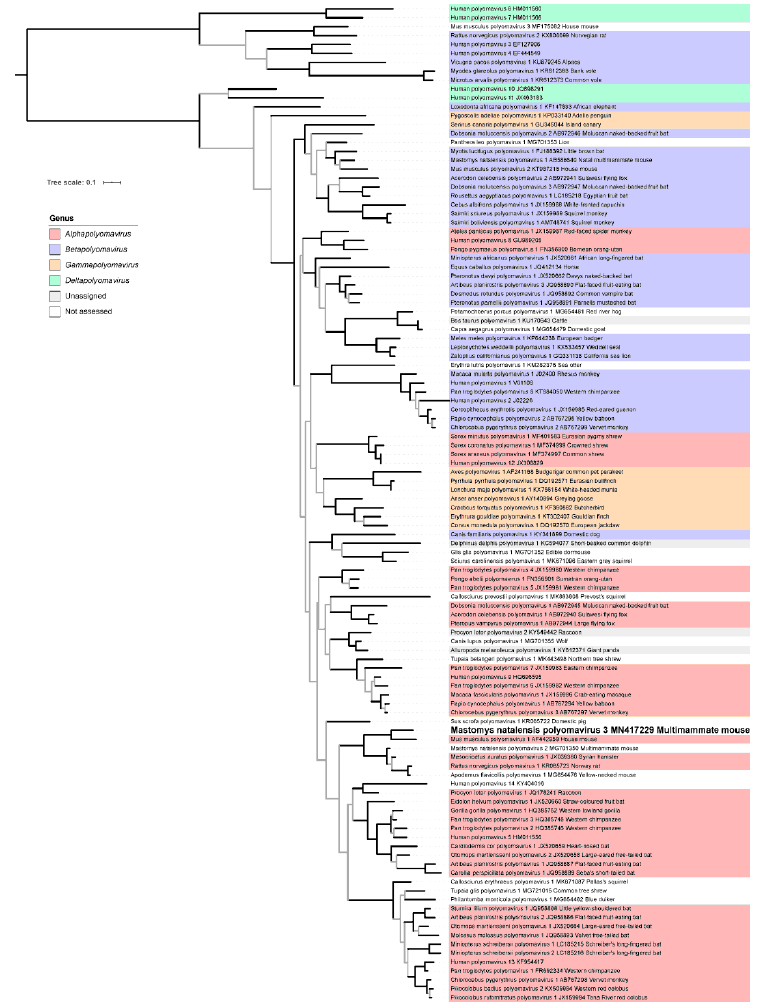

Supplement: Supplementary file 8 — ESM 8 Maximum-likelihood tree analysis of polyomaviruses based on VP1. Phylogenetic relationships of polyomaviruses, including the novel Mastomys natalensis polyomavirus 3, based on blocks of conserved amino acids in the VP1 sequence. For further details, see the legend of Figure 4. (PPTX 127 kb) [file 705_2020_4738_MOESM8_ESM.pptx]

## Slide 1
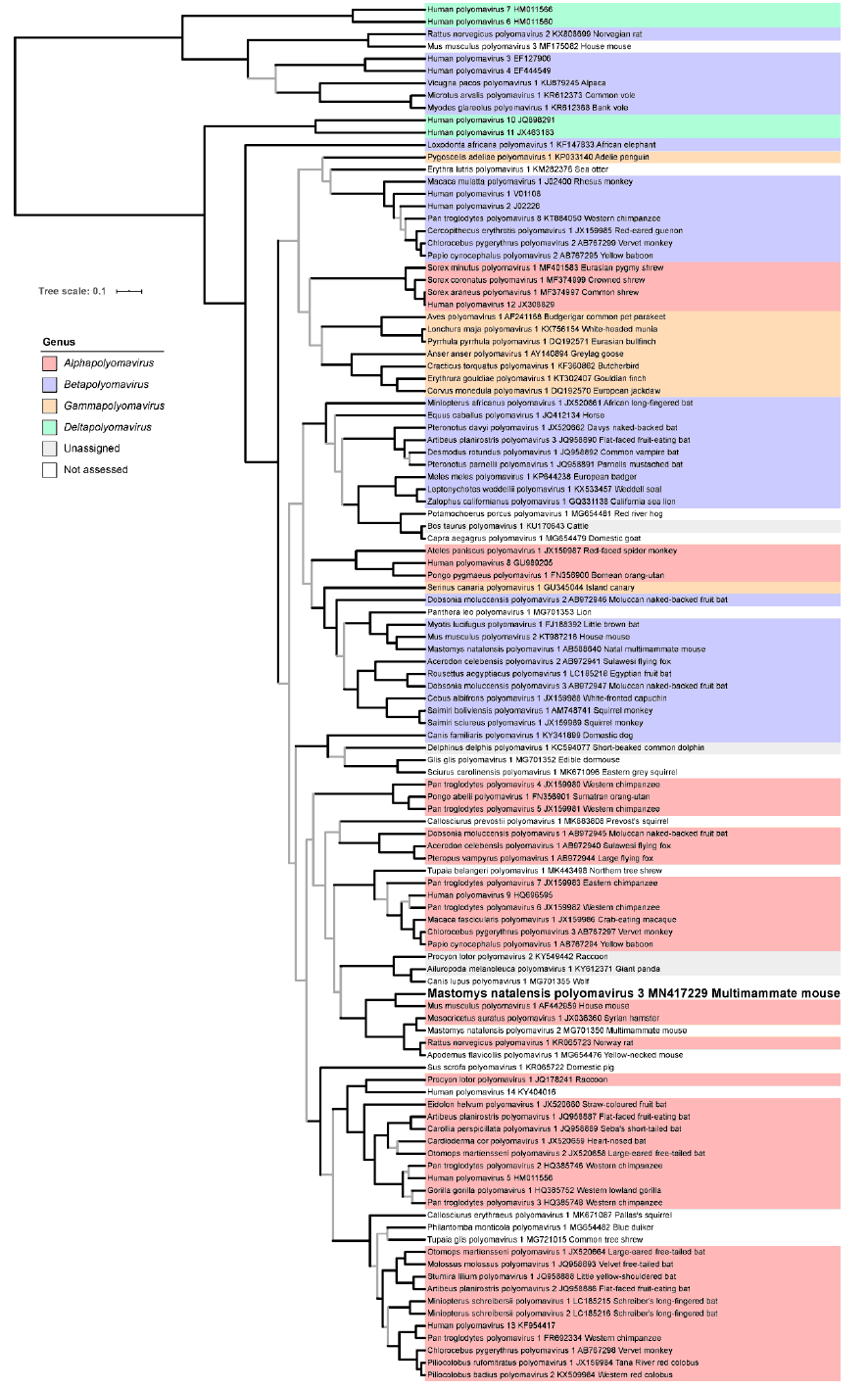

Supplement: Supplementary file 9 — ESM 9. Maximum-clade-credibility tree analysis of polyomaviruses based on VP1. Phylogenetic relationships of polyomaviruses, including the novel Mastomys natalensis polyomavirus 3, based on blocks of conserved amino acids in the VP1 sequence. For further details, see the legend of Figure 4. (PPTX 170 kb) [file 705_2020_4738_MOESM9_ESM.pptx]
